# Curation, integration and visualization of bacterial virulence factors in PATRIC

**DOI:** 10.1093/bioinformatics/btu631

**Published:** 2014-09-30

**Authors:** Chunhong Mao, David Abraham, Alice R. Wattam, Meredith J.C. Wilson, Maulik Shukla, Hyun Seung Yoo, Bruno W. Sobral

**Affiliations:** Virginia Bioinformatics Institute, Virginia Tech, Blacksburg, VA 24061, USA

## Abstract

**Motivation:** We’ve developed a highly curated bacterial virulence factor (VF) library in PATRIC (Pathosystems Resource Integration Center, www.patricbrc.org) to support infectious disease research. Although several VF databases are available, there is still a need to incorporate new knowledge found in published experimental evidence and integrate these data with other information known for these specific VF genes, including genomic and other omics data. This integration supports the identification of VFs, comparative studies and hypothesis generation, which facilitates the understanding of virulence and pathogenicity.

**Results:** We have manually curated VFs from six prioritized NIAID (National Institute of Allergy and Infectious Diseases) category A–C bacterial pathogen genera, *Mycobacterium*, *Salmonella*, *Escherichia*, *Shigella*, *Listeria* and *Bartonella*, using published literature. This curated information on virulence has been integrated with data from genomic functional annotations, trancriptomic experiments, protein–protein interactions and disease information already present in PATRIC. Such integration gives researchers access to a broad array of information about these individual genes, and also to a suite of tools to perform comparative genomic and transcriptomics analysis that are available at PATRIC.

**Availability and implementation:** All tools and data are freely available at PATRIC (http://patricbrc.org).

**Contact:** cmao@vbi.vt.edu.

**Supplementary information:** Supplementary data are available at *Bioinformatics* online.

## 1 INTRODUCTION

Virulence factors (VFs) are an important class of gene products that help pathogens to evade a specific host’s defensive mechanisms to establish infection in a specific environmental condition, thereby resulting in a disease state for that host. Studying VFs facilitates our understanding of pathogenicity and infectious disease mechanisms, and helps researchers to develop new methods for disease prevention and therapy (Casadevall and Pirofski, 2009; Keen, 2012; Onderdonk 1988; Wu *et al.*, 2008). There are a few existing VF databases that provide comprehensive collections of bacterial VF genes, such as VF database (VFDB; http://www.mgc.ac.cn/VFs), MvirDB (http://mvirdb.llnl.gov) and Victors (http://www.phidias.us/victors). These resources link published information about virulence directly to the genes or to protein families that contain the genes (Chen *et al.*, 2012; Zhou *et al.*, 2007). Other resources, like UniProt and KEGG (Kyoto Encyclopedia of Genes and Genomes), designate specific genes as VFs without necessarily showing the published support of this declaration (Ogata *et al.*, 1999; UniProt, 2011). Despite these resources, there remains a need to further identify VF genes, link them with published experimental evidence and integrate that data with the wealth of omics data that can reveal protein function, expression patterns or other information that show the gene’s role in virulence and the disease processes.

The Pathosystems Resource Integration Center (PATRIC) is the all-bacterial Bioinformatics Resource Center designed to support basic and applied biomedical research on bacterial infectious diseases (Gillespie *et al.*, 2011; Wattam *et al.*, 2014). PATRIC supports a variety of diverse data types such as genomics, transcriptomics, protein–protein interactions, 3D protein structures and DNA sequence typing data with their associated metadata. PATRIC also provides analysis tools for genome annotation, comparative genomics, transcriptomics, pathways, host–pathogen interaction and disease-related information (Driscoll *et al.*, 2011). Integrating bacterial VF genes is a logical next step in these data integration effort.

In the present work, we manually curated VFs from six prioritized National Institute of Allergy and Infectious Diseases (NIAID) category A–C bacterial pathogen genera (http://www.niaid.nih.gov/topics/biodefenserelated/biodefense/pages/cata.aspx), *Mycobacterium*, *Salmonella*, *Escherichia*, *Shigella*, *Listeria* and *Bartonella*, and integrated the curated information into PATRIC. In addition, we also integrated the VF information from VFDB and Victors into PATRIC. The resulting integrated information can be easily accessed and visualized through PATRIC’s Web interface. By leveraging the curated VFs, the information from both VFDB and Victors and the preexisting PATRIC resources, PATRIC’s VF module enables users to access the comprehensive omics, and host-pathogen disease information about the VF genes, perform comparative genomic and transcriptomics analysis for these VFs and store the analyzed information in their private workspace. The private workspace then becomes an important hypothesis-generating capability for bench biologists to advance VF knowledge and develop counter measures.

## 2 METHODS

### 2.1 Development of controlled vocabulary

We developed a controlled vocabulary to categorize VF genes and to enhance the global search capability available at PATRIC. The vocabulary was based on the combination and modification of the VF classifications and terms used by other databases, such as VFDB, MvirDB, COG and peer-reviewed literature (Chen *et al.*, 2012; Tatusov *et al.*, 1997; Zhou *et al.*, 2007). The major VF classification used included the following: adhesion, invasion, avoidance of host defense, extracellular growth, intracellular survival and replication, movement within host, acquisition of nutrients, regulation of gene expression, secretion systems, toxin and antibiotic resistance. The vocabulary terms used are listed at PATRIC Web site (http://patricbrc.org/portal/portal/patric/SpecialtyGeneSource?source=PATRIC_VF; under ‘Classification’ at the left panel).

### 2.2 VF genes

We collected bacterial VF genes from publicly available databases (VFDB, MVirDB and Victors) and from peer-reviewed publications. We searched the literature to identify new VF genes that are not included in the databases, and also to find primary references establishing the virulence of the genes identified at the other resources. The names of the VF genes, any available accession numbers, organisms studied and associated PubMed ID for each paper used were recorded.

### 2.3 Curation process

**Fig. 1.**
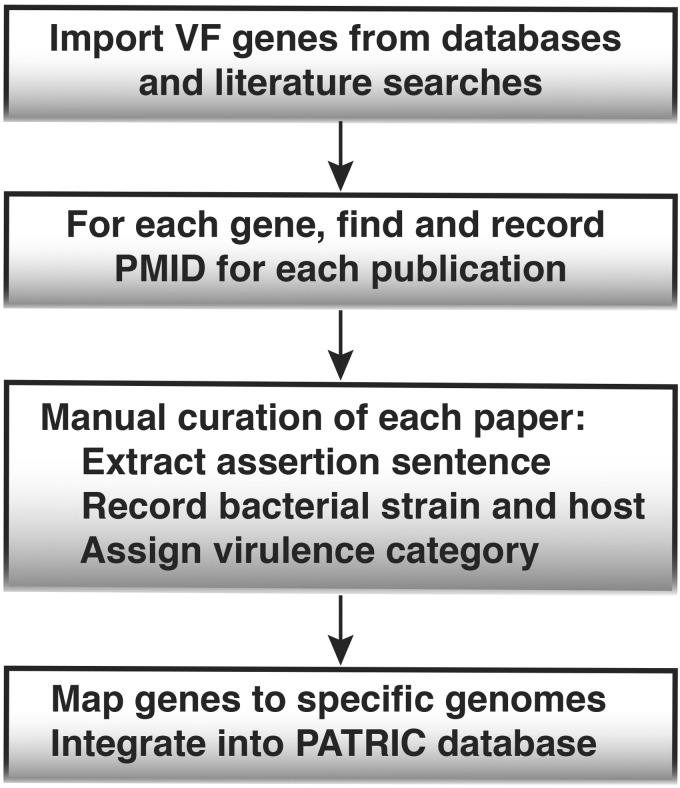
PATRIC VF gene curation workflow

The curation workflow is outlined in Figure 1. After initial collection of VF genes from other databases and peer-reviewed literature, curators followed the curation guideline to curate VFs using experimental evidences. First, for each VF gene in the list, a search for specific papers providing experimental evidence or computational function analysis about that gene was initiated. Emphasis was on finding direct experimental evidence of virulence on a specific host. However, if no experimental evidence exists, the computational analysis and the author’s description about the gene function are recorded. For each paper selected, sentences from the literature that assert the function of the VF gene studied as well as the name of the bacteria strain(s), the host(s) or cell types used in the experiment were extracted. Curators then assigned the VF gene with the defined categories (the controlled vocabulary as described above) based on the study. If the gene could not be classified into a specific category with the only evidence being a non-specific virulence phenotype, such as attenuation of a gene mutant strain, then the gene was assigned to a category denoted as ‘Virulence’. The extracted sentences that were chosen from the papers summarized the experimental evidence, virulence phenotypes or author’s description about the function for the VF gene(s) studied in the literature.

### 2.4 Mapping of curated VFs with PATRIC genes

Integration of the manually collected VF genes involved providing a direct mapping between genes that have been experimentally verified to genes present in the PATRIC database. Individual papers were analyzed with the specific object of acquiring the strain name of the bacterium used and identifiers for the gene used in the experiment. In an ideal situation, a gene’s GenBank accession number or RefSeq locus tag is provided (Pruitt *et al.*, 2012) and it directly maps the information from the publication to the gene and genome in PATRIC. When not available, other information, like the name of the gene, the gene symbol, a UniProt ID, the sequence of the gene, the neighborhood surrounding the gene in question or other cited papers were used to identify the gene used in the experiment. When the evidence was good, a direct map between the gene and genome specified in the paper and the identified gene at PATRIC was generated. These genes were identified as VFs in the PATRIC database, and were given an evidence label of ‘Literature’ on the PATRIC Web site. If despite all the efforts, a gene could not be verified, the associated literature data were discarded.

In addition to the manually curated VF genes, we integrated data from VFDB and Victors, including reference gene sets, their corresponding genomic sequence and all available literature. If sequence data were not available at the resource, the gene identifiers were used to collect corresponding sequence data from GenBank. These sequences were used to create a BLAST database of VFs. All genes in the PATRIC were searched against this database using BLASTP. The top BLAST hits for each genome were parsed and filtered based on the sequence identity and the sequence coverage using the following criteria: (%Query coverage ≥ 80 OR %Subject coverage ≥ 80) AND %Identity ≥ 80.

To identify homologs of these genes, the genes in each genome that had the top BLASTP hit against each of the genes in the VF database and met the filtering criteria were identified as potential VFs in the PATRIC database, and were given an evidence label of ‘BLASTP’ on the PATRIC Web site. This indicated that the gene was highly similar to a gene in either VFDB or Victors, or to the genes that had been manually curated. These, too, were given an evidence label of ‘BLASTP’ on the PATRIC Web site.

## 3 RESULTS AND DISCUSSION

### 3.1 Curation status

We curated 1572 VF genes from 1071 published papers for six prioritized NIAID category A–C bacterial pathogens. The number of curated unique VF genes and unique papers for each organism is listed in Table 1. As the VF curation is a continuing effort, we continue to update new virulence information for these organisms and are extending the curation work to other bacterial pathogens.

**Table 1.** VF curation status

Genus	No. genes	No. papers
*Mycobacterium*	537	311
*Salmonella*	496	355
*Escherichia*	251	101
*Listeria*	176	182
*Shigella*	86	102
*Bartonella*	26	20
Total	1572	1071

### 3.2 Integration of curated VF genes into PATRIC database

There were 2837 genes with direct literature references that were collected from Victors and 1572 from the PARTIC manually curated set. The PATRIC set contained some genes that mapped only to genes found in the RefSeq that were not called by the RAST annotation pipeline, which is used for annotation consistency across all the genomes in PATRIC (Overbeek *et al.*, 2014). Although there were 1572 genes curated, 1338 of these could be directly mapped to genes called by PATRIC. The unmapped genes were still kept in the PATRIC database with links out to their original resources. The combination of genes from Victors and the genes that could be mapped to PATRIC totaled 4175. As the same genes were sometimes identified by both databases, the total of unique genes with literature references was 3742. Each of these unique genes with direct references establishing their virulence was identified on the Web site with an evidence code of ‘Literature’, meaning that there was direct literature evidence linked to the gene in question. There were 1149 genes collected from VFDB, but this resource has a different method of curation where they link literature to a FIGfam and not directly to genes. Although these data are available in PATRIC, the evidence for these genes was listed as ‘BLASTP’, indicating that the identified gene was highly similar to one in the reference set, but lacked the direct literature evidence to merit the ‘Literature’ evidence code.

All protein sequences from each of these reference gene sets were used to create a BLAST database to which all the predicted genes from each genome at PATRIC were compared using the sequence identity and query coverage discussed above. These reference genes were mapped to 4 532 816 genes across the 79 934 557 genes annotated across the 21 640 genomes available in PATRIC as of July 2014. All of the genes that were identified by homology and not direct literature evidence were given a ‘BLASTP’ evidence code. Filters have been provided on the Web site that can adjust the level of BLAST homology, allowing researchers examine stringencies at a level of homology they are interested in.

### 3.3 Visualization of VFs in PATRIC

#### 3.3.1 Overview of the VF visualization

**Fig. 2.**
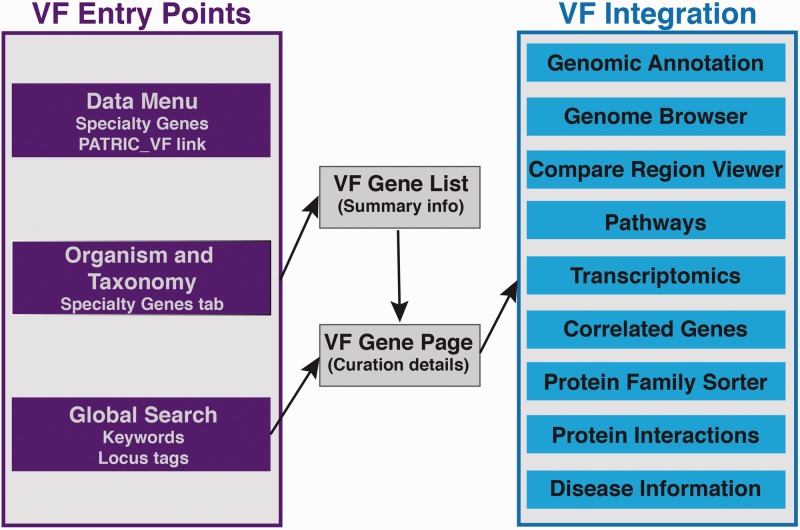
Visualization and analysis of VFs in PATRIC

The integrated VF information can be easily accessed and visualized through PATRIC Web interface. By leveraging the curated VF information with the existing PATRIC resources, the PATRIC VF module enables users to access the comprehensive omics data and tools. Figure 2 shows an overview of how VF genes can be accessed and visualized in PATRIC, and how other components in PATRIC with information, data and tools can be used for the VFs.

There are several entry points for accessing the VF genes: (i) The Specialty Gene page linked from the top DATA menu on PATRIC home page provides hyperlinks for all VFs curated in PATRIC (PATRIC_VF) and the VFs from VFDB and Victors; (ii) A ‘Specialty Genes’ tab is provided at each specified organism page and at any taxonomy level, which links to a list of VFs for that organism or level; (iii) The global search on any PATRIC page allows researchers to enter the VF gene symbol, ID or keywords to search for these genes, with the return being a list of VF genes that match the search criteria. Each gene returned in the search has a hyperlink to a more detailed gene page. As the VF genes are fully integrated with other components of PATRIC, users can easily gain access to other related information, data and tools from the VF gene page (Fig. 2). This page includes the direct evidence curated from the published literature, a view of the gene’s location in the genome (Genome Browser) or a conserved gene neighborhood (Compare Region Viewer), the ability to do comparative genomics and pathway analysis and also access to transcriptomics, protein–protein interactions, sequence typing and other data and analysis tools. Genes, or collections of genes of interest, can be stored in a private workspace accessible by the registered user.

#### 3.3.2 VF gene list and gene pages

**Fig. 3.**
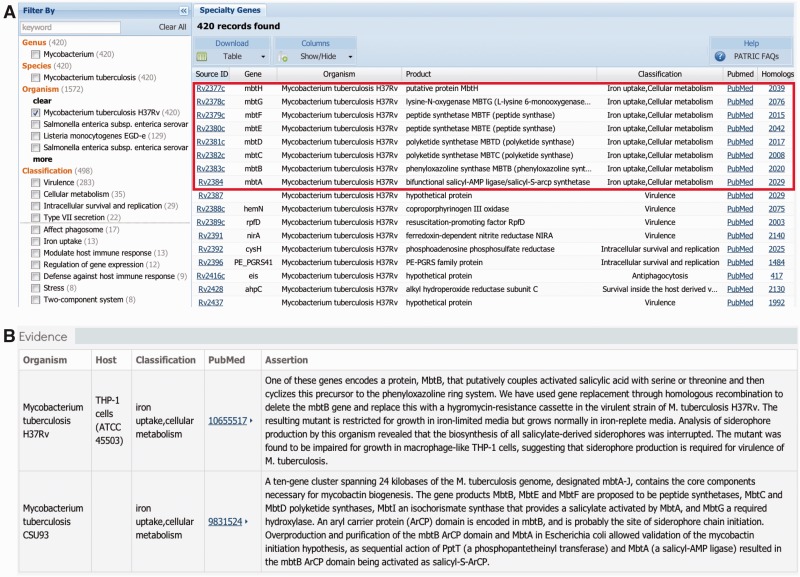
Visualization of VFs in PATRIC. (**A**) VF gene list with filtering options. (**B**) VF gene curation details

Figure 3A shows a list of PATRIC VF genes from the Specialty Genes search for *Mycobacterium tuberculosis* H37Rv. There are 420 VF genes curated in PATRIC for *M.tuberculosis* H37Rv. The left panel provides a filtering mechanism that allows users to select/filter on different organism(s) of interest and on different virulence classifications based on the vocabularies described in Section 2.1. The right panel is a table listing the selected VF genes. In this example, *mbtA-H* mycobactin biosynthesis genes are among the curated gene list. The table provides summarized information including the source ID, gene name and functional annotation, virulence classification, links to the PubMed papers establishing virulence and homologs of the gene. The Homologs column is hyperlinked to a list of homologs of the corresponding VF gene. PATRIC provides filters so that researchers can adjust the levels of homology to more stringent or more relaxed. Each homolog gene is labeled as ‘PATRIC_VF’ if there is direct experimental evidence from literature, or ‘BLASTP’ indicated as having possible virulence properties by homology. The source ID for each VF gene in the table links to a detailed gene page displaying the curated information from the literature. Figure 3B shows the portion of the VF gene page that describes the details from curation that include the bacterial strain tested, the host or cell type that was used, the virulence classification assigned by the curators and the assertion sentences extracted from the paper that provide the evidence of virulence.

### 3.4 Analysis of VFs using integrated omics information

One of the main advantages of PATRIC’s VF resource over other VF databases is the integration of rich omics data and analysis tools, giving a one-stop shop experience for researchers. Here we use *mbtB*, a mycobactin biosysnthesis gene in the *mbtA-J* gene cluster as an example to illustrate how the integrated omics information and tools can be used for this gene (De Voss *et al.*, 2000; Quadri *et al.*, 1998). An additional VF example is provided in the supplementary material. A hyperlink from the ‘Source ID’ of the VF gene on the detailed VF information page is provided for users to access more comprehensive information about this gene. In addition to the functional properties, several tabs are provided for this gene to access the Genome Browser, Compare Region Viewer, the pathways that the gene is involved in, interactions with other proteins, transcriptomics data and the correlated genes that have similar expression patterns.

#### 3.4.1 Genomics information and tools

The detailed gene page provides the mapped identification numbers and locus tags from different resources such as RefSeq, UniProt, NCBI, EMBL, etc., which enables easy access to different annotation sources of the same gene for comparison. The gene can be viewed in a Genome Browser, where it can be compared with other annotation sources. The Compare Region Viewer allows users to compare genomic regions around the gene of interest across other closely related genomes. Using this viewer, one can quickly detect differences in translation start sites, potential frame shifts or missing genes. In addition to the gene product annotation, other functional properties of the gene such as Gene Ontology, Enzyme Commission, FIGfam and pathway assignments, structure and protein–protein interaction information (if any) are also provided (Meyer *et al.*, 2009). Supplementary Figure S5 shows an example graph for protein–protein interactions. The DNA and protein sequences can be downloaded. If the virulence gene, e.g. *mbtB*, was also found in other VF databases such as VFDB and Victors, the information from these databases about this gene is also provided.

#### 3.4.2 Pathway analysis and tools

**Fig. 4.**
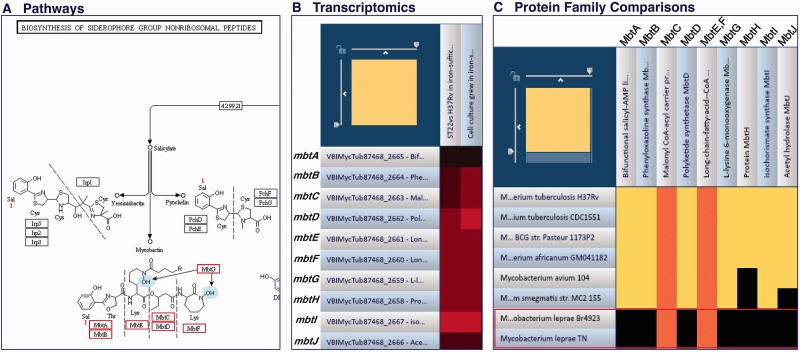
Analysis of VFs using integrated omics information. (**A**) Pathway map showing genes involved in biosynthesis of mycobactin (highlighted in red). (**B**) Heatmap view of expression profiles for the *mbt* gene cluster. (**C**) Comparative FIGfam analysis of *mbt* gene cluster; Cell color represents number of proteins from a specific genome in a given FIGfam. Black = 0, yellow = 1, orange = 3+

The ‘Pathways’ tab on the gene page provides a list of pathways in which the gene is involved. In PATRIC, three pathways include *mbtB*: Biosynthesis of siderophore group nonribosomal peptides, lysine biosynthesis and tryptophan metabolism. As *mbtB* is a mycobactin biosynthesis gene, the pathway summarizing the biosynthesis of mycobactin (Biosynthesis of siderophore group nonribosomal peptides) is one choice to look for the impact of this gene on metabolism and other genes involved in the pathway. The name of the pathway is hyperlinked to a Pathway Map page that has a tabular summary of the enzymes involved and the KEGG map for this pathway (Fig. 4A). The pathway map shows that in addition to *mbtB*, *mbtA*, *mbtC*, *mbtD*, *mbtE*, *mbtF* and *mbtG* are also involved in the biosynthesis of mycobactin (highlighted in red). Mycobactin biosynthesis is essential for the survival and virulence of *M.tuberculosis* (De Voss *et al.*, 2000; Quadri *et al.*, 1998; Reddy *et al.*, 2013). Without the functional *mbt* genes, mycobactin is not made and the iron uptake process is disrupted, which is critical for virulence. The pathway analysis can help one understand the biological function of the VF genes.

#### 3.4.3 Transcriptomics data and correlated genes

The ‘Transcriptomics’ tab on the gene page provides a list of available transcriptomics datasets for the gene of interest that has been added to the PATRIC resource. All comparisons in which this gene is differentially expressed are listed in a table along with the associated metadata, which includes strain, gene modification, experimental condition and time point. The summary of the datasets is presented graphically with bar graphs and pie charts (Supplementary Fig. S1). As of July 2014, there were 41 experiments and 453 comparisons available for *mbtB* gene expression data in PATRIC, one of which includes iron-dependent gene expression (Rodriguez *et al.*, 2002).

The ‘Correlated Genes’ tab shows a list of genes that have correlated expression profiles to the specified gene across all available transcriptomics datasets. Users can specify a correlation coefficient cutoff (between 0 and 1) and choose to view positively or negatively correlated genes. There are 53 correlated genes for *mbtB* based on all available transcriptomics data in PATRIC when using the default correlation coefficient value of 0.4. *mbtA-J* genes are clearly among these correlated genes for *mbtB*, which is consistent with the function of this gene cluster (Table 2). The correlated gene function in PATRIC is currently calculated in real time. Depending on the total number of available experiments of the gene being tested, the response time for displaying the result ranges from seconds to minutes. The result takes longer for genes from organisms such as *M.tuberculosis* and *Escherichia coli* for which correlation is computed based on expression of all genes across hundreds of experimental conditions.

**Table 2.** *mbtB* correlated genes

Gene	Correlation
*mbtB*	1
*mbtC*	0.9
*mbtD*	0.893
*mbtE*	0.858
*mbtF*	0.786
*mbtH*	0.734
*mbtG*	0.674
*mbtI*	0.67
*mbtA*	0.529
*mbtJ*	0.422

The PATRIC transcriptomics tools allow users to view the expression profiles of selected genes of interest in a heatmap view. Figure 4B shows an example of the heatmap view of expression profiles for the *mbt* gene cluster in an experiment examining the role of IdeR regulator in an iron-dependent gene expression (Rodriguez *et al.*, 2002). The iron-dependent transcriptional profiles of wild-type and *ideR* mutant strains were analyzed using iron-sufficient and iron-deficient conditions. The comparison clearly showed that all *mbt* genes were upregulated when comparing the wild-type strain in iron-sufficient condition with iron-deficient condition and comparing *ideR* mutant strain with wild-type strain under iron-sufficient condition. The result indicated that the *mbt* genes were regulated in a similar fashion by iron and the IdeR regulator. The transcriptomics analysis can help researchers understand the regulation of the VFs.

#### 3.4.4 Comparative FIGfam analysis of mbt genes

The Protein Family Sorter tool at PATRIC enables researchers to examine the distribution of specific isofunctional FIGfams, known as FIGfams, across different genomes (Meyer *et al.*, 2009). This tool can be used to compare the *mbt* FIGfams in mycobacterium genomes to see which genomes have or lack this gene cluster, or specific members within it. Figure 4C shows a 2D heatmap view of the FIGfam comparison among selected *Mycobacterium* genomes. It is evident that *Mycobacterium leprae* Br4923 and *M.leprae* TN (highlighted in red) lack the *mbtA–J* gene cluster, indicating that they do not have a mycobactin biosynthesis mechanism like *M.tuberculosis*. This is consistent with the previous findings that mycobactin was absent in *M.leprae* (Kato, 1985). *Mycobacterium leprae* uses an alternative mechanism for iron uptake process (Morrison, 1995).

#### 3.4.5 Between and within multi-locus sequence type comparative FIGfam analysis of VFs

The FIGfam sorter tool can also be used to look at within and between DNA sequence type (ST) VF differences in the multi-locus sequence types (MLSTs) incorporated in PATRIC as genome level metadata. This is useful for researchers who work with MLSTs and want to examine possible differences in disease phenotype between ST as well as what VF genes would make good candidates for expanding MLSTs by VF genes (Maiden, 2006). For instance, looking at the *E.coli* VF operons for iron uptake, fep and iuc, in the FIGfam sorter, it shows that there is considerable variation in the presence/absence of FIGfams for the fep operon between STs, whereas iuc has universal presence between STs, which indicates that iuc is a good candidate for including in a virulence MLST profile (de Lorenzo *et al.*, 1986; Pierce *et al.*, 1983). This finding is consistent with the published conclusions (Ngeleka *et al.*, 2002).

### 3.5 Comparison of PATRIC VF database with other databases

The presence of several databases devoted to VFs may beg the question of why yet another database is needed and how PATRIC VF resource differs from the others. UniProt and KEGG are valueable resources in their own right, and although they identify genes as VFs, they do not necessarily show the published support of this declaration. Victors is another powerful resource that provides literature, but it lacks integrations with other information. VFDB has a core dataset of 26 pathogenic genera that contain 459 VFs with 2059 associated publications. As in VFDB, PATRIC curators did extensive literature review in an effort to provide evidence of virulence, but, in PATRIC, this was targeted at finding direct experimental evidence of virulence that could be mapped to specific genes in their associated genomes, and not to a FIGfam as occurs in VFDB. In PATRIC, data collected from publications include the genome and gene, the host organism or cell type and sentences from the original article that supports an assertion of virulence. This allows researchers to see the supporting evidence without relying solely on the conclusions drawn by a curator. In addition, in an effort to integrate all types of useful information, the data from publications that document a lack of virulence in a known VF is also collected, as are the conditions (host, experimental factor) under which this occurred.

The type of integration available at PATRIC allows researchers to see virulence data in the context of comparative genomics, transcriptomics data and correlated gene expression, disease information or from the perspective of different sequence types (i.e. MLST). Having this variety of types of data available to a researcher in a single resource is what truly distinguishes PATRIC from other resources.

## 4 CONCLUSION AND FUTURE DIRECTIONS

Pathogenicity of bacteria continues to be an intense focus of research and funding. Knowing what specific genes have been tied to virulence is important, but being able to see the original data and experimental conditions, as well as having other information integrated with these data can be extremely powerful for the research scientist. PATRIC has made an initial foray in to this type of integration with curation initially targeted at six bacterial genera with plans to expand across of the NIAID category A–C priority pathogens. In addition to manually curating data associated with virulence, we have computationally integrated data from two VF resources, allowing researches to see the homologs of all genes identified as virulent across the 22 000 genomes currently in PATRIC. Specific PATRIC tools allow individual researchers to adjust the degree of homology (expressed in percent coverage or percent identity of the BLASTP hit), to apply limits to the stringency used to establish homology. In addition, the integration of VFs in PATRIC provides an access to all the other data and tools available for analysis, making it a valuable experience for researchers and a unique resource.

## Supplementary Material

Supplementary Data
