# Supplementary material for: Curation, integration and visualization of bacterial virulence factors in PATRIC
Source: Bioinformatics. 2014 Sep 30;31(2):252–8. doi: 10.1093/bioinformatics/btu631 (PMC4287947; doi:10.1093/bioinformatics/btu631)
Supplement: Supplementary Data [file supp_31_2_252__index.html]

Curation, integration and visualization of bacterial virulence factors in PATRIC — Curation, integration and visualization of bacterial virulence factors in PATRIC — Curation, integration and visualization of bacterial virulence factors in PATRIC — Supplementary Data 

# Curation, integration and visualization of bacterial virulence factors in PATRIC

## Supplementary Data

files

**Files in this Data Supplement:**

- Supplementary Data - docx file
- Supplementary Data - docx file
