# Supplementary figures and images for: Curation, integration and visualization of bacterial virulence factors in PATRIC
Source: Bioinformatics. 2014 Sep 30;31(2):252–8. doi: 10.1093/bioinformatics/btu631 (PMC4287947; doi:10.1093/bioinformatics/btu631)

**
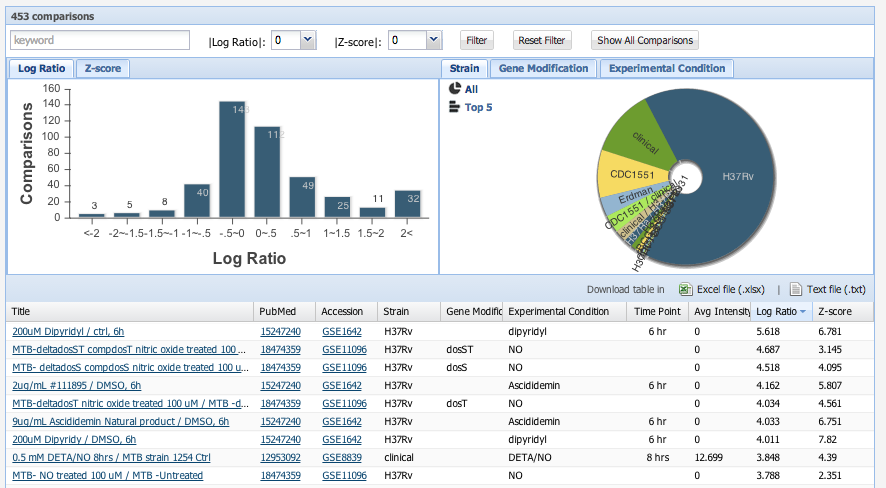
**

**Figure 1S.**Transcriptomics data for *mbtB* gene.

Supplement: Supplementary Data [file supp_btu631_Suppl_material_1.docx]
