# Supplementary material for: Curation, integration and visualization of bacterial virulence factors in PATRIC
Source: Bioinformatics. 2014 Sep 30;31(2):252–8. doi: 10.1093/bioinformatics/btu631 (PMC4287947; doi:10.1093/bioinformatics/btu631)
Supplement: Supplementary Data [file supp_btu631_Suppl_material_2.docx]

**An Additional Example of a PATRIC Curated Virulence Factor**

*inlB* (Imo0434), an internalin gene from *Listeria monocytogenes* EGD-e, is an important virulence factor for bacteria invasion. The URL of the curated information for this gene is <http://patricbrc.org/portal/portal/patric/SpecialtyGeneEvidence?source=PATRIC_VF&sourceId=lmo0434>. The VF gene page shows the curated information about the bacterial strain tested, the host or cell type that was used, the virulence classification assigned by the curators and the assertion sentences extracted from the paper that provide the evidence of virulence. We curated multiple publications that provide the direct experimental evidences for the involvement of *inlB* gene in the invasion process. Figure 2S shows the portion of VF gene page listing the curated details about *inlB* gene.


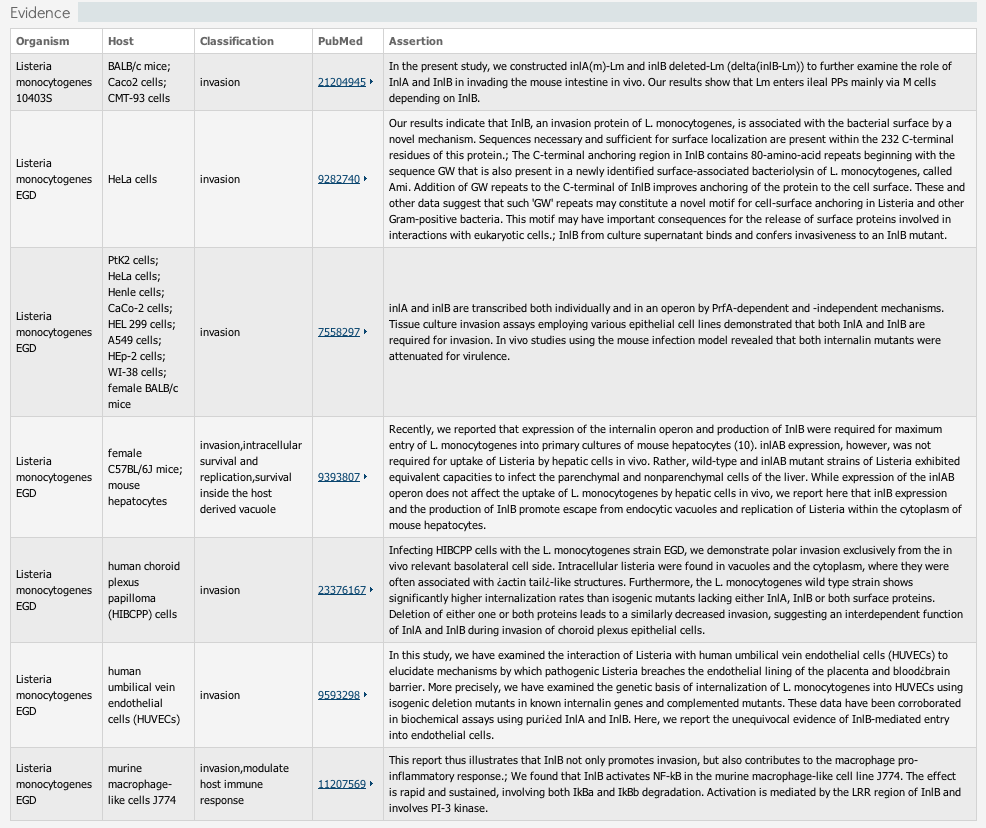


**Fig. 2S.** VF gene curation details for *inlB*.

The “Transcriptomics” tab on the gene page provides a list of available transcriptomics datasets for *inlB* gene (Figure 3S). As of July 2014, there were a total of 33 experiments and 129 comparisons available for *inlB* gene expression data in PATRIC.

**
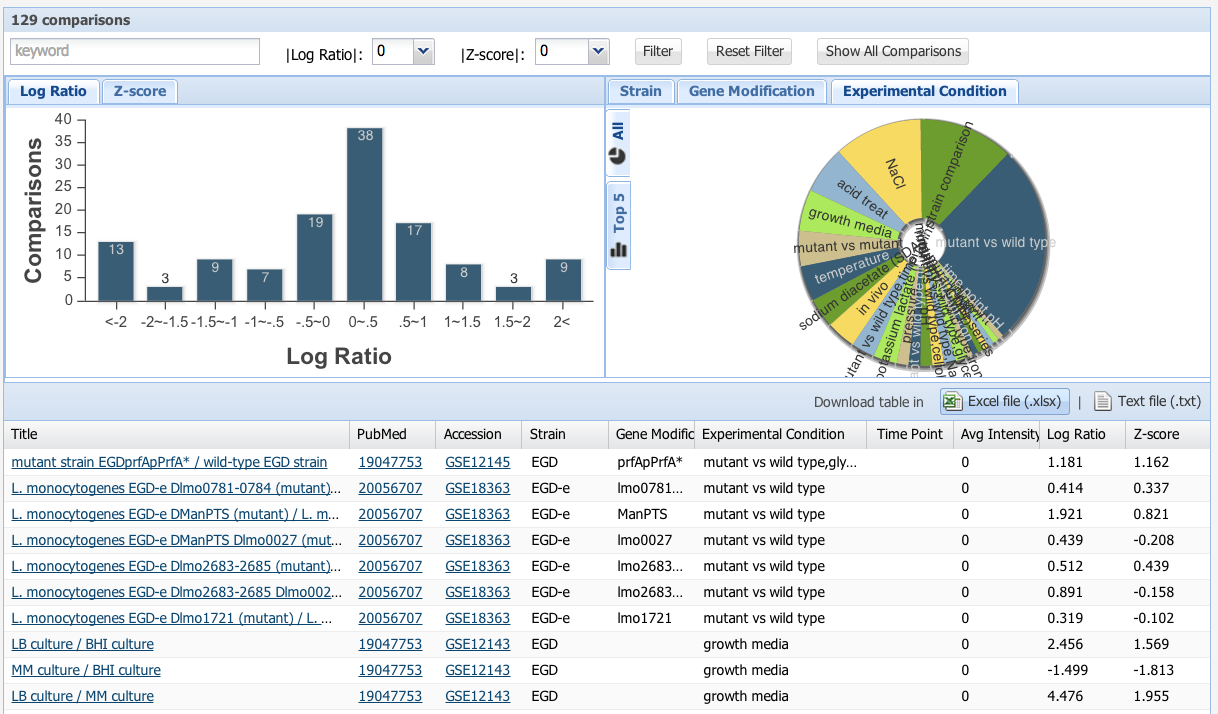
**

**Figure 3S.**Transcriptomics data for *inlB* gene.

There are total of 46 correlated genes for *inlB* based on all available transcriptomics data in PATRIC when using the default correlation coefficient value of 0.4. Figure 4S shows the top ranked genes that are correlated with *inlB*. Clearly, *inlA* (Imo0433), another internalin gene in the *inlAB* operon is highly correlated to *inlB*. Interestingly, some VF genes such as *plcB* (Imo0205), *inlC* (Imo1786), *actA* (Imo0204), *hly* (lmo0202), *plcA* (Imo0201), and *prfA* (Imo0200) are also correlated well with *inlB*.


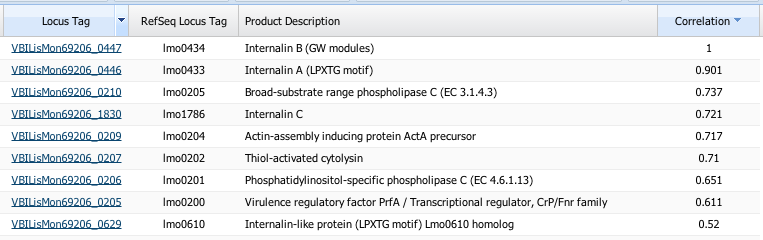


**Figure. 4S.** *inlB* correlated genes.

Figure 5S shows protein-protein interaction information for *inlB*. The table below the interaction graph listed three direct evidences showing the direct interactions between *Listeria* InlB protein and human hepatocyte growth factor.


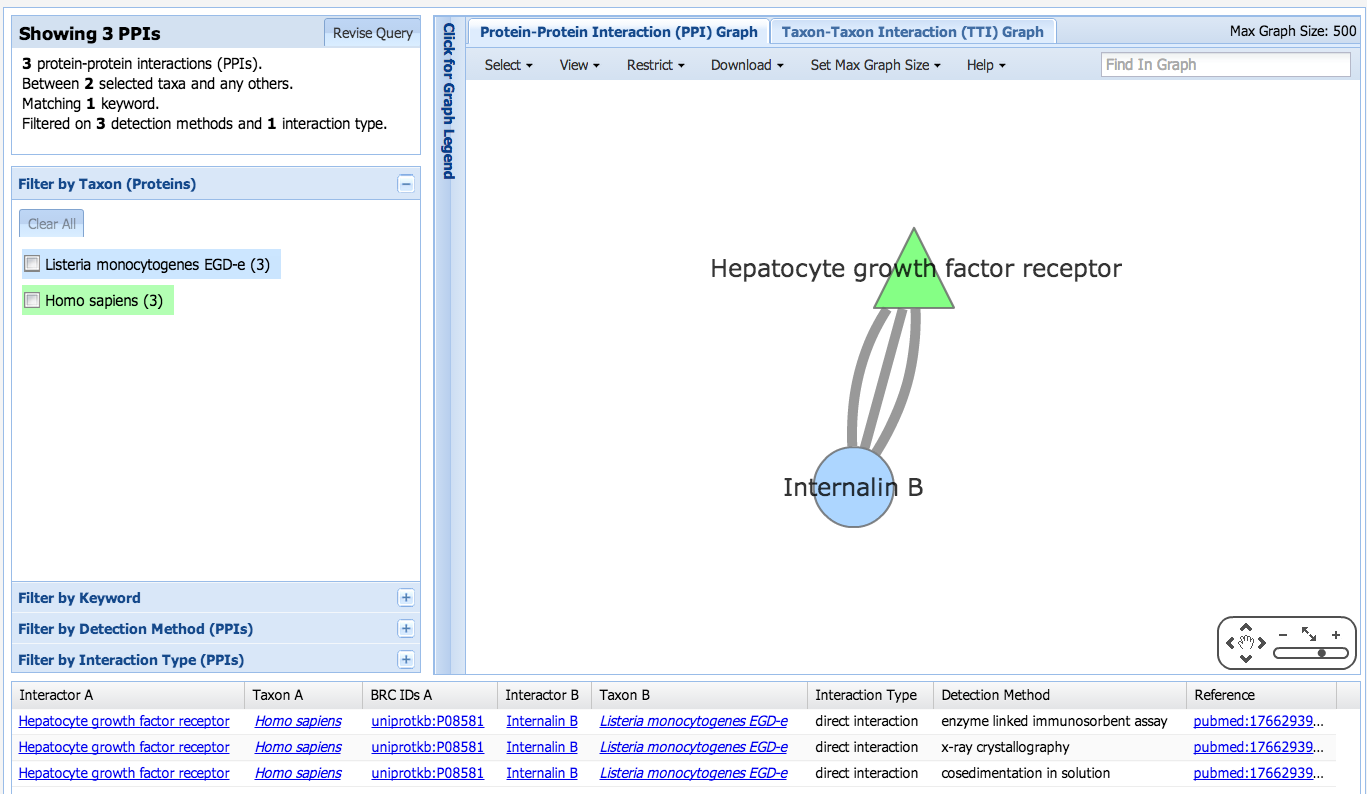


**Fig. 5S.** Protein-protein interactions for *inlB*.
